# Lesson Enactments: Maintenance in Everyday Educational Practice

**DOI:** 10.1007/s42438-023-00401-z

**Published:** 2023-04-13

**Authors:** Sara Mörtsell

**Affiliations:** 1 Faculty of Education and Business Studies, University of Gävle, Gävle, Sweden; 2 Department of Education, Umeå University, Umeå, Sweden

**Keywords:** Teaching, Lessons, Maintenance, Actor-network theory, Digital technology, Covid-19

## Abstract

This article explores lesson enactments as co-constitutive of human-technology relationality in everyday schooling, rather than neutral backdrops for educational activities. In doing so, the article introduces maintenance as its key concept, drawing on insights from maintenance studies and actor-network theory (ANT). Being both theoretically and empirically informed, maintenance means reconsidering lessons, and digital technologies, as part of lively and vulnerable objects achieved in sociomaterial practices and not merely stable in function and use. The empirical case of lesson enactments comes from fieldwork with an upper secondary school in Sweden during Covid-19. The article analyses situations of maintenance with online class calls and scheduling meetings. Herein, lessons turn into a topic of concern and mechanisms of maintenance enact educational order and prevent disorder. The article demonstrates how putting maintenance to work articulates and identifies so far neglected and mundane practices with digital technology in education. In light of this, the article argues for recognising maintenance in educational practice as too long overshadowed by use, reinforced by a persistent user-technology dichotomy. Finally, the article discusses how maintenance invites reconsiderations of the dominant before-after debate that the Covid-19 pandemic attracts and calls attention to the mundane maintenance of lessons regardless of breakdowns.

## Introduction

This paper explores lessons as sociomaterial assemblages in the everyday of schools. Frequently overshadowed by issues of planning, design and resourcing, lessons have tended to take up an unreflected and taken-for-granted container position for educational activities as merely marking the passing of time. The rise of sociomaterial approaches has shown this to be an inadequate account of the vibrant role of matter in education (Decuypere and Broeck 2020; Decuypere and Simons 2016; Edwards and Fenwick 2015; Fenwick and Landri 2012). Building on these insights, lessons are here addressed as intricately holding educational order together and, at the same time, becoming enacted of the day-to-day practices of education. Specifically, the paper explores lessons at an upper secondary school in Sweden during the Covid-19 pandemic and brings attention to the mundane and relational efforts that enact lessons in this setting. Working resolutely with Microsoft Teams and with preventing disorder, lessons at the school were elusive, liable to change and constantly negotiated. The features of Teams—online calls, the course feed and a scheduling form—describe functions of the technology, yet, as this paper will show, enact lessons in more unpredictable ways than their functions promise. These enactments, in which lessons are made and make educational practice, are the concern of the study. To address it, the study draws on maintenance studies in science and technology studies (STS) (Denis 2020; Denis et al. 2016; Denis and Pontille 2015, 2023; Jackson 2019; Russell and Vinsel 2018) to explore technologies and lessons as vulnerable and lively situated at the intersection of routine and breakdown. Bringing maintenance to education and lesson enactments opens up avenues of inquiry in which digital functions and objects are not assumed to be stable and naturally enduring.

The explorations of lesson enactments in this paper pay particular attention to the concept of maintenance in relation to actor-network theory (ANT) (Law 2009; Law and Singleton 2005; Mol 2010). Firstly, this is a recognition that maintenance, for this paper, is an ANT-informed and empirical notion of the ‘sticky combination of adaptability and perseverance’ (Mol 2008: 91). Secondly, ANT offers a theoretical starting point in sociomaterial vulnerability rather than taking vulnerability to be the deviant state from normalcy. Herein, technologies and objects are never fully functioning nor fully broken but assumed to be always relationally enacted into being (Law and Singleton 2005). This relationality is productive for maintenance since it is alert to vulnerabilities, which will be further outlined below. For education, this is a potentially generative domain away from a persistent binary reading of digital technology. It opens up for acknowledging vulnerabilities and neglect rather than fixating on mastery, competence and use (cf. Mol et al. 2010; Puig de la Bellacasa 2017). Thus, the significance of maintenance for how lessons and digital technologies take part in education deserves both theoretical and empirical foregrounding. It generates questions about what is maintained in everyday education and the specificities of the mechanisms involved, here in the case of the pandemic.

The aim is to explore the mechanisms of an ANT-informed notion of maintenance within education, both theoretically and empirically. By connecting maintenance studies to education and technology research, a set of neglected practices in education performed specifically in relation to lessons and digital technology is addressed. Drawing on fieldwork during Covid-19, the focus is on how lessons are enacted and what lessons enact to maintain educational orders in pandemic education. The exploration is guided by the research question: How are lessons enacted on a daily basis at this school? The paper concerns theorising how lessons, as educational orders, become provisionally shaped and brought into being, rather than determining whether lessons, maintenance or, indeed, technologies are effectively working or not.

This paper is organised as follows. First, I outline previous research from maintenance studies and stress some emerging stories of maintenance in research on higher education during the pandemic to identify mundane lesson enactments as a generative domain for human-technology relationality. The next two main sections serve the argument that maintenance is theoretically and empirically informed. The first of these presents an ANT account of the theoretical contributions of maintenance studies that are deployed in the study. The second section presents the empirical setting of an upper secondary school in Sweden before analysing lesson enactments at the school. The article concludes with a discussion on the implications of maintenance for education research that addresses three related binaries that maintenance in lessons unsettles: user-technology, breakdown-routine and before-after.

## Maintenance and Digital Technology in Education

To situate the paper, this section outlines maintenance studies as providing the grounding for exploring lesson enactments. I also present how the paper responds to pandemic education by relating the taken approach to more dominant ones.

The interdisciplinary field of maintenance studies engages with the material care practices that tenaciously check, repair and restore order by keeping failures, disorders and decay at bay or to a minimum. It is ‘the distinctive forms of work that go into keeping things the same’ (Russell and Vinsel 2018: 7). Concerning digital technologies, maintenance studies have empirically explored material care and repair such as breaking smartphones (Jackson 2019), outdated software (Cohn 2019) and electronic waste (Callén and Criado 2016). For the case of education and technology, Rosner and Ames (2014) report on a case study of One Laptop Per Child (OLPC) from Paraguay. Founded by Nicholas Negroponte, the OLPC program idealised children’s ability for learning by repairing and failed to predict the material wear and tear of computers in schools, including the costs of repair, and the range of negotiations that maintenance involved in the local setting. Similarly in connection to education, Vandenabeele and Decuypere (2022) examine repair cafés as sites of public pedagogy in acknowledgement of the work in fine-tuning practices of humans and material objects. Sharing a concern for vulnerable technologies and things, these empirical explorations attest to maintenance and repair practices’ insistence that there is no ‘single use’ (Vandenabeele and Decuypere 2022). Having long been neglected practices in most descriptions of technology, including undesirable technologies, this study provides additional insight into mechanisms of maintenance by exploring lesson enactments and the mundane efforts to keep the everyday of school going.

Moreover, maintenance studies raise critical issues about what counts in knowledge practices. When neglected and largely invisible work of dealing with mess and monotonous upkeep is brought into the theoretic frame of technology, educational engagements with technology that were erased can take shape. To address this, the paper draws on the theoretical contributions articulated by Denis and Pontille in a number of works on maintenance as ontological interventions in the mundane life of things that is often devalued, or even denied, under an instrumentalist logic (Denis 2020; Denis et al. 2016; Denis and Pontille 2015, 2020, 2023). In turn, they build on scholarship in ANT that suggests technologies always do more than what is expected of them and that ‘things are just as unpredictable as people’ (Mol 2008: 50). For education and technology research, maintenance studies suggest both empirical and theoretical relevance of mundane and day-to-day practices with digital technology. Firstly, the empirical insights from maintenance studies invite education research to challenge instrumental user-technology logic as the only relevant human-technology relationality. Secondly, the theoretical work done by maintenance studies identifies mundane routines like lesson enactments as a generative domain in educational practice for exploring this relationality. The theoretical contributions will be further outlined in the next section on how ANT accounts for maintenance as unfolding in practice. In sum, maintenance is an empirically and theoretically informed concept.

As mentioned above, the setting for the lessons explored in this study is the intersection of breakdown and routine located at an upper secondary school during the Covid-19 pandemic. From the onset of Covid-19 in early 2020, the dominant framing of *emergency remote teaching* has become widely adopted and emphasises the need for separating improvised teaching from established modes of well-planned online teaching (e.g. Hodges et al. 2020; Moore et al. 2021; Rapanta et al. 2021). Although this approach has been timely and prolific, it also means that a lot of the work on pandemic educational practices has been premised on separation rather than relation. This is addressed here by attending to relationality enabled by maintenance and ANT, which aligns with other relational approaches to the pandemic-education encounter such as posthumanist and feminist materialist theory (Gravett et al. 2021; Murris 2020).

Furthermore, stories of maintenance in higher education have appeared in ANT-informed work on the pandemic and inspired this study to pay greater attention to the mundane efforts in schools in similar encounters with difference, change and sameness. One story of maintenance is offered by Alarcón López and colleagues (2021) who report on improvisations and doing things ‘on the fly’ for the examinations in higher education to function well. Here is a familiar rationale of ‘the show must go on’ that reinforces conventional actors and with improvisations that assemble new actors, e.g. the internet provider. The case of examination shows that form-keeping maintenance is, at the same time, form-giving, i.e. transformative (Denis 2020). In addition, Gourlay (2022) raises another, during Covid-19, well-rehearsed and confounding phrase of ‘it is just not the same’ on teaching with platforms such as Zoom or Teams. In other words, when ‘the show must go on’, the show necessarily also changes, and ‘it is just not the same’. The intimate familiarity of the two phrases highlights that maintenance practices during the pandemic can be regarded as vast and accessible in educational practice and therefore deserving of scholarly attention.

Postdigital scholars, who insist on messy heterogeneity of the digital (e.g., Jandrić and Knox 2022; Macgilchrist 2021; Mörtsell and Gunnarsson 2023), offer additional reasons for taking a relational approach with mundane lesson enactments. The above-mentioned elusiveness of lessons with unpredictable digital technology raises questions about how such indeterminacy should and can be taken into account. Mess, stickiness and uncertainties are relational processes that sit well with ANT’s fostering of sensibilities towards difference and mess (Law 2009; Law and Singleton 2005; Mol 2010). Additionally, I will go on to develop the argument in this paper that maintenance is empirically and theoretically comfortable with mess. Maintenance gives meaning to the concern that if things (lessons, spaces, online calls, materials, bodies) are conveniently working well in education, what mundane day-to-day maintenance practices may have been put aside from that analysis? How and where do they need to be studied? These concerns situate this study to contribute explorations of maintenance in an encounter with education.

## An ANT Account of Maintenance

So far, I have mentioned vulnerable technologies and the liveliness of matter as central to maintenance studies. This assumption can be further articulated with an ANT-informed relational ontology and specifically with the two notions of performativity and multiplicity, which were excluded in the early ANT literature (Law 2009). This section stages those developments of ANT for theorising maintenance and lesson enactments.

For ANT, all things mutually constitute each other, i.e. perform, in material-semiotic webs of entangled relations (Law 2009; Mol 2010). If an object, such as an online meeting in Teams or a lesson, is performing as definite, well-functioning and stable, it is the relational effect of distributed and materially heterogeneous practices. This performativity is in a reciprocal relation at the same time enacting the practices, which means that practices are world-making. So too are lesson enactments. This ANT principle of performativity in relation to maintenance suggests that reality and material order is not passively waiting to break or be maintained, and materiality is not inert, but rather brought into being of precarious sociomaterial enactments (Denis and Pontille 2015, 2020; Jackson 2019).

There are two theoretical contributions that maintenance studies have generated that are of note to this paper, specifically how they are qualified with ANT. As a first theoretical contribution, maintenance studies have brought to the fore that insofar as there are robust and stable versions of things doing their job they are co-constituted of vulnerability and decay. In that sense, material resilience and durability seize to be taken for granted as matters of fact and become matters of concern (Latour 2005). In maintenance practices, any working order and, what we might think of as, disorder are constant and inseparable versions of each other and, consequently, there is no totalising or singular stable order of the world (Denis 2020; Denis and Pontille 2015; Jackson 2019). This resonates with the assumption of multiplicity that multiple versions and orderings of reality simultaneously are in flux that can be traced to the ANT notion of ontological politics (e.g. Mol 1999, 2002). Puig de la Bellacasa’s (2017: 44) question elucidates this: ‘what worlds are being maintained and at the expense of which others?’ With this assumption of multiplicity, practices such as research, description and maintenance are not innocent but become performative of versions of reality. With a relational ontology, nothing pre-exists its relations but is enacted *of* them in certain provisionary ways (Law 2009; Mol 2010).

The second and related theoretical contribution generated in maintenance studies is the questioning of a linear relation of maintenance and order. Close examinations into the mundane care of things and maintenance practice have articulated that order does not always follow as borne out of maintenance doings (Denis 2020; Denis and Pontille 2020; Jackson 2019). This connects with the long-standing interest that ANT scholars have directed at how a specific sociomaterial order manifests in *ordering* processes with other orders. This means that, for an ANT study, the focal point is not primarily on what sociomaterial orders there are, but on the work that temporarily achieves an assembled order. In theorising this, disorders and events in which orders are at stake harbour ambivalence and ambiguities as they are understood as presenting another order in co-existence (Law 2009; Mol 2010). In turn, disorder and mess have informed maintenance as a concept that bundles orders, i.e. hold versions of reality apart or together in tensions and overlaps. In the maintenance of subway signs, Denis and Pontille (2015: 355) find stable and vulnerable versions of signs in which ‘[o]ne version is completely dedicated to the other: stability does not emerge via the negation of vulnerability, but rather by its being taken into account’.

Given the two ANT notions of multiplicity and performativity outlined here, practices, events and relations become vital to exploring maintenance. Empirical cases of maintenance, Denis and Pontille (2020) point out, have been made possible and generated these theoretical developments. The developments are closely associated with ‘post-ANT’ literature and the concern ‘how to know’ mess (e.g. Law 2009; Law and Singleton 2005). Vice versa, earlier versions of ANT were unable to make room for maintenance and analytically excluded it from socio-technical accounts (Denis 2020). Importantly, being unfolded in practice means that maintenance as a theoretical concept informs and enriches the ANT repertoire with ‘a mode of engaging with the world’ that opens up potentialities (Mol 2010: 262). Being part of the ANT repertoire, maintenance addresses the vastness of what presents as mundane and independently ongoing, also in the encounter with education and technology.

## Method, Setting and Empirical Engagements

In line with methods in maintenance studies, the empirical orientation of ANT has motivated this study to ethnographically engage with situated and empirical events of maintenance in everyday of education caught in pandemic ambiguities of breakdown and routine. Based on being a regular upper secondary school in the Swedish context, the setting had been purposefully selected for a first study during the first wave of Covid-19 (see Mörtsell 2022). With the onset of the second pandemic wave, this study draws on 6 months of ethnographic engagements with the same school from December 2020 to the end of the term in June 2021. Located in a rural part of Sweden with high-speed connectivity, the school provided students with school laptops. Three of the teachers from the first study, teaching the same groups of students in different subjects, agreed to have the daily activities of eight of their courses followed with a guest account in the school’s Microsoft Teams application. The courses were in higher education preparatory programmes and taught to 63 students, aged 17–19, who I had met on previous visits to the school. The continuity with the same group of teachers and students allowed for establishing rapport (Beaulieu 2017). After introducing the project of following lessons, I checked in with each student individually for informed consent and again in specific situations such as interviews and recordings. In addition, the project had been approved by the Swedish Ethical Review Agency.

The empirical engagements with following lesson enactments in the pandemic combined modes of ethnography on digital technology and visits to the school (Beaulieu 2017; Burrell 2017). It involved regularly taking part in the online class calls when the teacher and all students on the course met in videoconference. As not all lessons had online class calls, engaging with each course’s ‘Team’, for example, the feed that gathered posts on communications and assignments became important. In the 41 online calls I took part in, I often stayed on the call talking to the teacher after students had left and sometimes used the chat. Thirteen students and six members of staff including the three teachers who taught the eight courses were interviewed. About halfway through the fieldwork, in April 2021, the local circumstances of the pandemic changed, making it possible to travel and visit the school. In combination with continuing with the guest account in Microsoft Teams and the online calls, lessons during five full-day visits were followed. To minimise physical contact and avoid crowding inside and outside of the school, the student cohorts had alternating days scheduled for lessons at school. The fieldwork comprised field notes, audio recordings, transcripts, screencasts, timetables and photographs.

In keeping with ANT methodologies, the analytic method put to work in this study is tracing. Mol (2010: 255) makes clear that the point of tracing is ‘not to tame the world theoretically’ but to make ‘so far unspoken events’ sensible. Because ANT is not an explanatory framework, tracing operationalises the ‘showing how’ of relational enactments in education such as lessons, rather than settling on ‘telling that’ they are relational (Decuypere 2019; Decuypere and Simons 2016; Fenwick and Landri 2012). Consequently, what other approaches might find a weakness becomes a strength in ANT, its adaptable repertoire that does not predict (Law 2009; Mol 2010). This means that limitations to the methodology include not seeking to explain the social or *give voice* to human actors (Beaulieu 2017). Instead, tracing performs with empirical events that are made to happen by the specificities of sociomaterial relations and entities. Inspired by maintenance studies, lesson enactments are here traced and explored in situations of maintenance, in which the working order of lessons is turned into a topic, a concern (Denis 2020). Understood this way, the empirical events are not merely representing themes or examples of the general aspects of fieldwork. Analytic questions for tracing are: how are ordering processes taking place with lessons at this school? How are order, disorder and other tensions stabilised to hold lessons and the everyday of education together? What mechanisms of maintenance can be identified?

## Exploring Lesson Enactments with Maintenance

For the lessons to work on a daily basis, including the research engagements with them, considerable attention was directed at Microsoft Teams. Especially the feed for each course, chat messages, the digital scheduling form and the online class calls were engaged in enacting lessons. This section of the paper is organised in two parts, each beginning with an empirical event of lesson enactments followed by elaborations on the mechanisms of maintenance that the event and setting raise.

### Online Calls, Replications and Improvisations

The first empirical event is from an online class call on Microsoft Teams with the teacher Cecilia and a group of 15 students and me. Pseudonyms are used. All of us take part from home. We are just about to leave the call as Cecilia has given the instructions for the rest of the scheduled lesson and the next lesson the following day, and there is suddenly some confusion.

Klara: Ok, so we don’t have a call tomorrow even though it says so?

Cecilia: Oh really? [laughs] I’m sorry. No, you’ll work with your assignments, and no, we don’t have a call.

Josefine: Can’t we connect on a call anyway? I get more done if I can sit like that.

Cecilia: No problem. I’ll be right here in front of my screen as always. And, if we have a call, I might not get so many messages in the chat. It’s easier just to take questions directly on the call. It’s a really good idea, thanks. If you want, we can do that right now? I’ll stay on the call if anyone wants to stay on it until we finish?

The students say ‘bye’ and quickly leave one by one so that I am left with Cecilia on the call (Field note 2021-02-04).

With this event, mundane relationalities emerge on how material arrangements, replication and improvisations are co-performative of lessons. Klara checks in with the instruction posted on the feed. It is a trivial practice easily excluded from what counts in accounting for the function of the feed in this event. In relation to the material arrangement of bodies sitting/lying down in front of screens, the Teams feed becomes a sociomaterial actor with the capacity to attract our bodies and all our attention. The feed’s capacity to act is a relational effect of checking, bodies, screens, and homes, rather than an inherent feature. In this opening part of the event, the feed is not an independently ongoing object but rather enacted of feed practices. Even though there are some questions about tomorrow’s lesson, the arranged attention maintains the function of the feed and enacts stability to the technology as holding together (Mol 2010).

However, the planning of lessons become destabilised by Josefine’s articulation to ‘connect to a call anyway’. Here, the challenge of ‘a call anyway’ enacts the lessons as concerns. In relation to this concern, mechanisms of replication and improvisations set in. Josefine’s suggestion can be understood as a call to replicate a familiar educational order, namely lessons in classrooms. With a representational logic, it would count the online class call as a ‘better representation’ of lessons than leaving the call and continuing the lesson without it. However, with ANT, representation and codified meanings are not the point. Instead, replication is performative. Whereas replication and replacements have been accused of being non-innovative modes of digital education, maintenance studies have addressed the re-inventive character of such practices (e.g. Denis and Pontille 2015). By putting maintenance to work with this event, it is possible to acknowledge mechanisms of replications, of re-assembling parts, as generative. The replication mechanisms emphasise the relatedness of lesson enactments as multiple. In one and the same move, the ‘online call anyway’ relates to a stable educational structure of classroom enactments, and it does not. Replication enacts a set of relations in which objects ‘both changes and stay the same’ (Law and Singleton 2005: 338). The event thereby highlights lessons as repeated and performative doings and precarious achievements, rather than rapid and singularised decisions and choices (cf. Mol 2008).

Concerning lesson enactments in the event, planning and improvising lessons ‘on the fly’ in the event become co-ordinated with maintenance. In playing with the online call’s functions so that a call without a necessary ‘caller’ can also work and enact lessons, the digital functions and attributes become contested and vulnerable to change. Improvising with the digital technology opens up for what an online class call can be. As mechanisms of maintenance, improvisations ‘always overwhelm the standardized procedures’ (Denis and Pontille 2015: 355). Nevertheless, when confronted with the leave meeting button, the planned-improvised lesson is not achieved as the students abruptly leave the call. The online call is constantly accessible, yet in the encounter with lessons, the call appears only constantly so in inaccessible ways. Not achieving the lesson in practice demonstrates the surprise of ‘how technologies do not live up to their promise’ (Mol 2008: 43). The lesson becomes enacted as moulded with ambivalence. The event addresses how mechanisms of improvisations and play enact the online meeting in Teams as dependent on the function of ‘a caller’ for it to work well and operate. At the same time, it involves the inabilities to accommodate the spontaneity of lessons and educational practices.

The event raises the matter of how mundane and even trivial form-keeping and form-giving practices enact lessons. It shows how lessons are stabilised by keeping and maintaining shape with replication. However, this event shows how stability is achieved in relation to keeping other orderings at bay—doubts on what to do, unresolved questions and the struggle to get work done. In that way, the maintenance situation harbours and discloses co-existing order and disorder (Law 2009). Furthermore, understood as a situation of maintenance means that lessons are enacted of both following instructions and doing things ‘on the fly’ to prevent disorder and make stable other orders. Maintenance studies insist that improvisations should not be rationalised into failed planning (Denis et al. 2016). In these overlapping practices of planning-improvising and replication-innovation, mechanisms of replication and improvisations are easily overlooked, yet they are significant to lesson enactments.

#### Scheduling Lessons and Adaptability

**Fig. 1:**
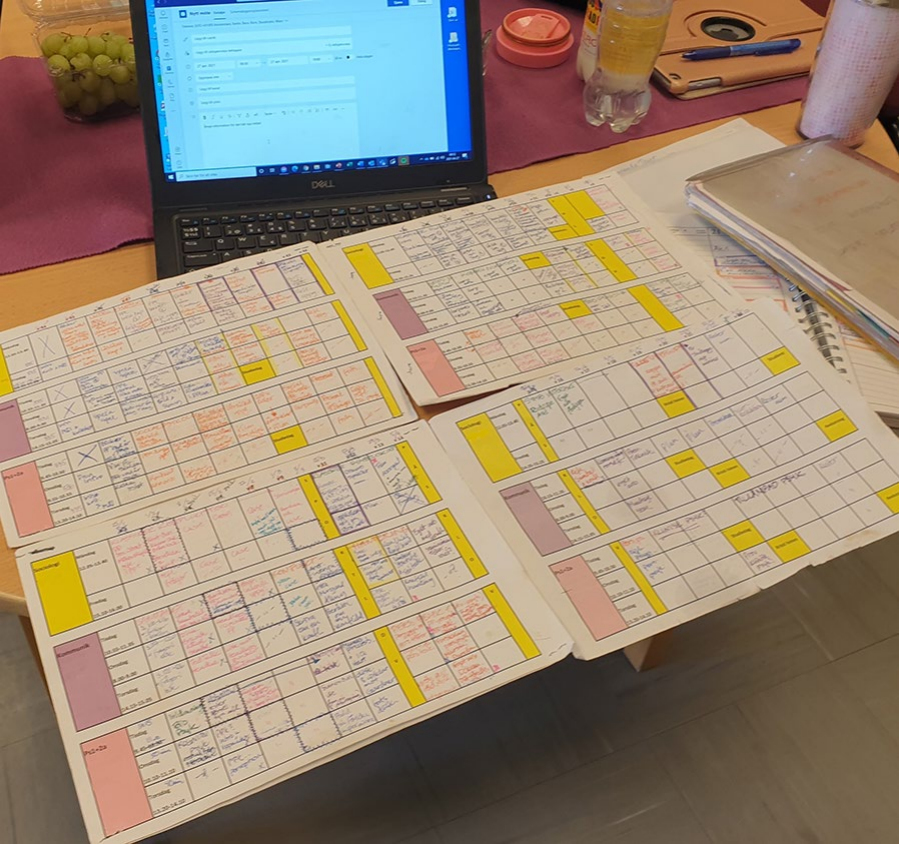
A scheduling event

Thus far, lesson enactments at the school have been described in relation to online class calls as not straightforward. Online class calls only lasted a portion of a lesson, and some lessons were organised as independent work and did not have any class calls. Perhaps lessons were not as important as other things. While lessons seemed to be losing shape, they were, nonetheless, far from ignored. A dominant setting for this was that lessons were constantly being scheduled. In the courses I participated in, the scheduling meant that the teachers manually entered a post in Teams for each online class call. Importantly, it served a digital function of the technology since the scheduling post generated the join meeting button—the online call demanded a joining function to work well. Oftentimes, the teachers also entered posts for lessons without a call only to clarify there was none for a particular lesson. Sometimes the posts were entered shortly before a lesson was due to start and other times weekly, which is what Cecilia did, usually on a Monday. The teachers called this novel task ‘to create meetings’. Being in the margins of both teaching and lessons, the constant scheduling was easily ignored and overlooked, yet this neglect is precisely what invites scheduling to contribute to the exploration of maintenance in lesson enactments. Figure 1 is a scheduling event in which lessons become the topic of a maintenance situation.

The scheduling event as a situation of maintenance evokes questions about what allow for lessons to hold together on a daily basis. In scheduling practices, lesson enactments are the achievements of many different and distributed actors. Firstly, scheduling is the relational effect of the distribution of sites, the local specificities of the oversized year plan with jotted words and the global digital standards, displayed on the computer screen in the scheduling form where details are entered. The year plan presents the capacity for overview enacted by attachments such as the curriculum, the school’s timetable and the changing pandemic protocols. Each attachment is work-intensive, distributed and, in this site of pandemic school life, enacted as safe and reliable. The school’s Covid-19 protocol advised everyone to persevere by sticking to the timetable no matter what the pandemic emergencies might bring. The timing of lessons was remarkably static, and the school’s official timetable did not budge despite many creative ideas on how adjustments could alleviate stress. The protocol, then, is not merely a description of measures but performative of those measures. Understood as maintenance, scheduling enacts lessons as educational order, such as knowing what to do and when. At the same time, scheduling also works to maintain the digital function of the technology by allowing for the joining of class calls. Importantly, there are reciprocal movements, namely as teachers adapt to and take on the scheduling task, these components enact scheduling as a maintenance practice new to the school and transformative to the everyday practices. Putting maintenance to work with this empirical event demonstrates how scheduling enfolds both perseverance and adaptability.

But secondly, it is relevant to linger a while with how lessons were enacted here. Scheduling meetings as maintenance doings were not merely scheduling but became involved in performing lessons. How did a novel maintenance task like ‘create meetings’ also change and adapt lessons? Maintenance studies have shown that the linear maintenance-order representation, of scheduling something to take place in an orderly fashion without affecting what takes place, is not given (Denis 2020). Rather, in this mechanism of maintenance where lessons meet meetings, the technology addresses lessons in a particular way. For example, the digital scheduling form comes with its world of business-informed productivity tools that, in the encounter with education, maintains a certain office logic, a mode of ordering (Mol 2010). This logic is managerial in character and enacts meetings that address a particular (office) worker to call them. The above event with the online class call highlighted the online calls’ dependency on such a caller as unaccommodating to lesson spontaneity. Offices have little recognition for educational objects such as timetables and lessons. Thus, both the timetable and lessons become adapted to the technology, albeit with different effects. Being a reinforced and reliable actor, as described above, the timetable becomes further amplified in relation to its neglect in the digital technology. The timetable did not change. Lessons, on the other hand, became imposed on by the technology in ways that translated lessons into meetings. It raises the question if online meetings and lessons ‘are just not the same’ (Gourlay 2022), how do they relate?

So, language changes with scheduling but is it a matter of conversion as lessons travel between sites, i.e. the lesson in the year plan and the meeting in the digital scheduling form on the screen? Instead of looking for singularity, that one becomes another one, ANT advocates multiplicity and maintenance bundles multiple orders. If online meetings and lessons are ‘just not the same’, it is because they are irreducible to each other but still in practice co-ordinated. Online meetings, configured of digital on-screen joining and leaving buttons, offer little room for improvised and spontaneous interactions that enact lessons. The task of ‘creating meetings’ thereby maintains the digital technology and the everyday of pandemic education, but at the expense of lessons. In relation to scheduling, lessons gain the capacity to adapt and become subservient. Mol (2010: 256) insists that actors are not necessarily heroes but ‘may also seek to serve the world around them’. Since all objects and things are vulnerable, leaky and liable to change, adaptability cannot be understood as a property of lessons or meetings here. ANT suggests that they are different versions that hold together because ‘they flow into one another’ (Law 2009: 152; Law and Singleton 2005).

With the new teacher responsibility of scheduling meetings, mechanisms of adaptability are achieved, and lesson enactments come and go. In this setting of pandemic and educational practice, lessons are enacted as expendable to educational order (cf. Puig de la Bellacasa 2017).

## Discussion and Maintenance in Day-to-Day Education

In answering the question of how lessons are enacted in the everyday of education in this setting, the study has explored mechanisms of maintenance and re-assembled them as improvisations, replication and adaptability. They are mechanisms with which maintenance addresses lessons as repeated and performative doings and precarious achievements. In the analysed events, the achievements of order are called into question and multiple orders are at stake. Putting maintenance to work theoretically and empirically emphasises that for the everyday of education to stay the same in terms of lesson enactments in the pandemic, lessons needed to change. It is a case in which maintenance proves comfortable with change and sameness. Law and Singleton (2005: 339) articulate this as practising ‘make do and mend’. Lessons are, not surprisingly, enacted in many practices, of which this study has empirically explored the practices of online class calls and scheduling meetings. The implication is that with these empirical events conceptualised in the ANT repertoire with multiplicity and performativity, maintenance becomes a vital ‘co-ordinating device’ (Mol 2010: 266) of the continuing efforts in schools.

Exploring lesson enactments this way with maintenance means acknowledging that educational practice is enacted of entanglements of breakdown and routine also when emergencies are over. Maintenance studies have re-evaluated the dominance of breakdowns and innovation-centrism in STS and sociotechnical study (Denis 2020; Denis et al. 2016; Denis and Pontille 2023; Russell and Vinsel 2018). On the matter of the breakdown metaphor, Denis et al. (2016) argue that it privileges use (rather than maintenance). This article draws attention to this in two ways. Firstly, the dichotomy of breakdowns risks amplifying a dominant binary reading of technology that education repeatedly attracts—does the technology work or not? With maintenance, on the other hand, vulnerabilities are a shared concern that overcomes getting caught up in binary readings of technology. Instead, vast and familiar educational practices become theorised and noticeable and can be attended to. Maintenance sides with neglected practices and makes them stronger in the face of erasure (Mol et al. 2010). Secondly, maintenance as explored here with lesson enactments reaches beyond the educational breakdown setting of the Covid-19 pandemic. Whereas a major event like the pandemic insists on attracting a linear structure of ‘before and after’, in education further reinforced by proposals of *emergency remote teaching*, maintenance calls for watchful co-ordination that ‘cannot be normalized’ (Denis and Pontille 2015: 355). To the mundane routines of lesson enactments, breakdowns are not irrelevant, rather they are taken into account. Thus, maintenance as a theoretically and empirically informed concept raises the pressing matter of not one-sidedly, perhaps passively, waiting on breakdowns to occur. The fabric of (school) life calls for maintenance regardless of breakdowns (Mol 2008; Puig de la Bellacasa 2017).

As the liveliness of lessons and technology calls on maintenance, the interdependent relation of use and maintenance becomes foregrounded. Theorising maintenance with ANT makes possible an expansion of use relations in education that takes into account use as closely dependent on maintenance and multiplied in educational practice. User and used are mutually constituted in entangled webs. In other words, use relations are not singular or unidirectional (cf. Vandenabeele and Decuypere 2022). In lesson enactments, this study demonstrates how users and digital technology are infolded in maintenance practices. Thus, engaging with the concept of maintenance in this educational setting brings to light neglected and multiple nuances of use relations. Maintenance invites future education research to take a wider interest in use relations and ask what actors in education are made heroic.

To conclude, putting maintenance to work with lesson enactments calls into question claims of immutable objects and technologies in education. The importance of digital technology in the case of pandemic education is not caused by any superior digital or online features. If there is convenience and stability, as often associated with everyday digital technology, it is an effect of mundane and ordinary relations and practices. Speaking to that in this study are the enfolded mechanisms of maintenance—perseverance in adaptability, improvisations in planning and innovation in replication. Hence, bringing maintenance to the study of educational practices stages an intervention in the erased work overshadowed by the user-instrument logic by adding neglected descriptions (cf. Puig de La Bellacasa 2017). It invites considerations of how digital technologies present education with a wide range of situations of maintenance and responsibilities in the becoming of things such as lessons.
